# Aligning action across major infectious diseases in a dynamic global health landscape

**DOI:** 10.1371/journal.pgph.0006237

**Published:** 2026-04-02

**Authors:** Nathan Ford, Tereza Kasaeva

**Affiliations:** Department of HIV, TB, Hepatitis and STIs, World Health Organization, Geneva, Switzerland; PLOS: Public Library of Science, UNITED STATES OF AMERICA

**Fig 1:**
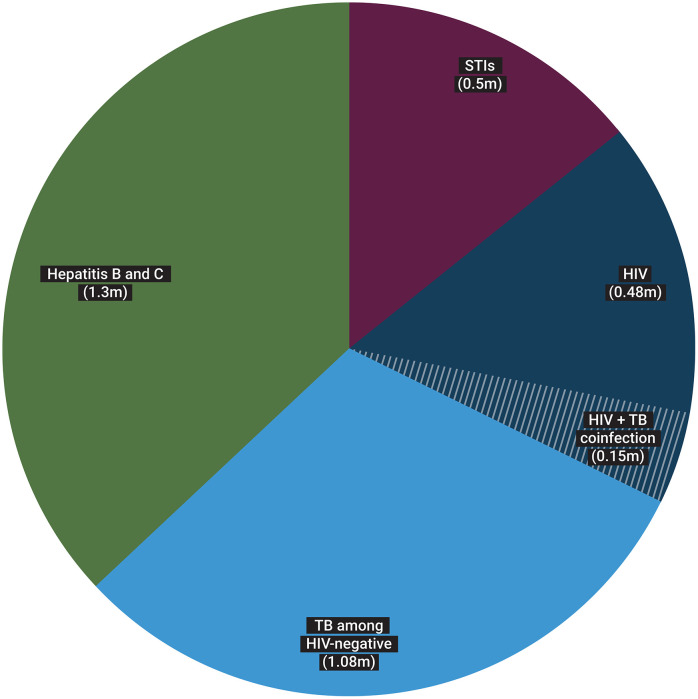
Deaths among people with HIV, TB, hepatitis B & C and sexually transmitted infections in 2024.

In July 2025, WHO merged its Department of HIV, Viral Hepatitis and STIs with the Global Programme on Tuberculosis and Lung Health, creating a new structure – the Department of HIV, TB, Hepatitis and STIs. Although this decision was driven by unprecedented financial shortfalls, there is a strong underlying rationale, creating opportunities to identify synergies and efficiencies without losing the gains made in recent years. HIV, TB, Hepatitis and STIs account for around 3.5 million deaths each year (Fig 1) [1,2].

The integration of services for HIV and tuberculosis has been a consideration since the beginning of HIV programmes in resource-limited settings. Early observations showed a rapid rise in tuberculosis incidence in countries with high HIV prevalence, with many tuberculosis programmes facing challenges to manage the expanding caseload [3]. As HIV treatment access was scaled up, opportunities for integration at primary care were assessed and pilot programmes reported successful outcomes [4].

Viewed from the perspective of HIV programmes, the merging of HIV and TB programmes make a great deal of sense. TB was recognized as a common co-infection among people living with HIV since the 1980s [5]. The public health response to HIV was inspired by TB programmes to standardize treatment regimens and monitor progress [6]. People living with HIV are about 14 times more likely to develop TB disease compared to all people diagnosed with TB, and it is the leading cause of hospitalization and death in this group, accounting for a quarter of HIV-associated deaths overall [1,7]. A number of core collaborative TB/HIV activities are proposed by WHO [8], and guideline recommendations to test for HIV, screen for TB disease, and provide TB preventive therapy have all been widely adopted.

From the perspective of TB programmes, the rationale for integration is context specific. In some parts of southern Africa, where more than half of incident TB cases are among people living with HIV, the benefits of an integrated approach are clear. Globally, however, less than 6% of all incident cases are people among people living with HIV. TB infection is driven by many other factors, and nine in ten TB deaths are among HIV negative individuals [1].

Hepatitis and STIs share common routes of transmission with HIV, and together with TB they all share common social and economic determinants of diseases, leading to substantial overlap in terms of populations most at risk. Groups at heightened risk include people who inject drugs, men who have sex with men, sex workers and their clients, pregnant women, and adolescents and young adults; migration, incarceration, poor nutrition and limited access to healthcare contribute to increased risk of infection and disease progression. Across all diseases, children have specific diagnostics, treatment and care needs requiring specialized expertise and services. The *Triple Elimination Initiative* to eliminate mother-to-child transmission of HIV, syphilis and hepatitis B remains a key priority for WHO to reduce burden of these diseases in children [9].

Adopting a unified approach to these conditions promotes integrated, people-centred service delivery, which is critical for reaching underserved and marginalised populations who bear the greatest burden. Integrated delivery of key interventions for these diseases is gaining momentum, led by countries facing a high burden of co-infections [10]. In 2024, 82% of individuals newly diagnosed with TB has a documented HIV test result highlighting strong programmatic linkage [1]. Among the 27 WHO focus countries for viral hepatitis that reported data on service integration, over 70% indicated that hepatitis B and C testing and treatment services are integrated within existing HIV services, while over 60% reported integration into primary care [2].

A core function of WHO is to deliver scientific guidance, normative leadership, and technical assistance to its Member States. The Department of HIV, TB, Hepatitis and STIs is committed to developing normative guidance aimed at reducing the incidence, mortality and burden of these diseases. Such normative guidance will promote the use of integrated diagnostic platforms [11], therapies that are compatible across diseases - including long-acting formulations [12], - and the alignment of service delivery approaches to support sustained care across conditions [13].

WHO’s normative work is informed by another core function: to collect, analyse, and disseminate reliable information on the global burden of disease to guide international health action. Through its coordinating role in global health, WHO compiles standardized data from Member States, produces global health statistics and mortality estimates, monitors trends in diseases and risk factors, financing and access to health services, and publishes epidemiological surveillance reports that inform the actions of governments, researchers, civil society and international partners. WHO will continue to guide global action and support countries to strengthen their surveillance systems to provide integrated collection and reporting of essential health data across HIV, TB, Hepatitis and STIs, working closely with other UN agencies and partners.

The bringing together of major infectious diseases at WHO headquarters reflects a reorientation that is underway in countries. In 2023 Member States committed to integrating the screening, prevention, treatment and care of TB and related conditions—including HIV, viral hepatitis, undernutrition, mental health disorders, and non-communicable diseases—within primary health care, including community-based services [14]. Practical country-level examples include integrating antiretroviral therapy refills with family planning and care for HIV-exposed infants in South Africa, incorporating screening and management of hypertension and diabetes into antiretroviral adherence clubs in Kenya, and aligning antiretroviral therapy refills with TB preventive therapy in Zambia and Zimbabwe. Recent evidence from Tanzania and Uganda suggests that integrated community-based care can be as effective as facility-based care for managing HIV, diabetes, and hypertension [15]; such evidence is critical to informing the scale-up of integrated care across health system.

As the post-2030 agenda for TB, HIV, viral hepatitis, and STIs takes shape, integration should be positioned as a transformative strategy for building resilient, people-centred health systems that advance broader development goals. Achieving this will require strong political commitment and global coordination. Preparations for the UN High-Level Meetings on HIV (2026) and TB (2028) provide key opportunities to mobilise leadership, sustainable financing, and enduring commitments to equity and health for all.
